# Magnon Torque Transferred into a Magnetic Insulator through an Antiferromagnetic Insulator

**DOI:** 10.3390/nano11112766

**Published:** 2021-10-20

**Authors:** Zhiren Chen, Zehan Chen, Xiaotian Zhao, Baoshan Cui, Hongnan Zheng, Lin Liu, Wei Jia, Tianhui Li, Zhixiang Ye, Mingxia Qiu, Ning Wang, Lei Ma, Hongyu An

**Affiliations:** 1 College of New Materials and New Energies, Shenzhen Technology University, Shenzhen 518118, China; 20183280072@stumail.sztu.edu.cn (Z.C.); 20183280060@stumail.sztu.edu.cn (Z.C.); 20183280028@stumail.sztu.edu.cn (H.Z.); 20183280033@stumail.sztu.edu.cn (L.L.); 2070413017@stumail.sztu.edu.cn (W.J.); 2110413006@stumail.sztu.edu.cn (T.L.); yezhixiang@sztu.edu.cn (Z.Y.); qiumingxia@sztu.edu.cn (M.Q.); 2 Shenyang National Laboratory for Materials Science, Institute of Metal Research, Chinese Academy of Sciences, Shenyang 110016, China; xtzhao@imr.ac.cn; 3 Songshan Lake Materials Laboratory, Dongguan 523808, China; cuibaoshan@sslab.org.cn

**Keywords:** magnon torque, magnetic insulator, antiferromagnetic insulator, spin-orbit torque

## Abstract

Electrical spin-orbit torque (SOT) in magnetic insulators (MI) has been intensively studied due to its advantages in spin-orbitronic devices with ultralow energy consumption. However, the magnon torque in the MIs, which has the potential to further lower the energy consumption, still remains elusive. In this work, we demonstrate the efficient magnon torque transferred into an MI through an antiferromagnetic insulator. By fabricating a Pt/NiO/Tm${}_{3}$Fe${}_{5}$O${}_{12}$ heterostructure with different NiO thicknesses, we have systematically investigated the evolution of the transferred magnon torque. We show that the magnon torque efficiency transferred through the NiO into the MI can retain a high value (∼50%), which is comparable to the previous report for the magnon torque transferred into the metallic magnet. Our study manifests the feasibility of realizing the pure magnon-based spin-orbitronic devices with ultralow energy consumption and high efficiency.

## 1. Introduction

**Figure 1:**
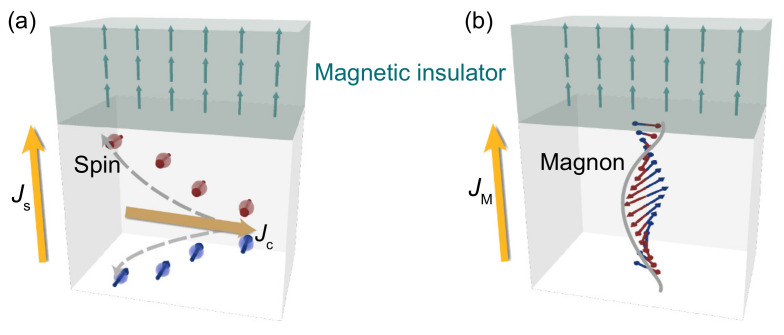
Schematic of the MI magnetization manipulated by (**a**) the electrical SOT via the electrical spin current $J_{s}$, and (**b**) the magnon torque via the magnon current $J_{M}$.

Discovering novel phenomena and functionalities originating from the spin-orbit coupling (SOC) is an emerging direction in spin-orbitronics [1,2,3,4]. In the spin-orbitronic devices, a pure spin current is generated from a charge current through the SOC, defined as electrical spin current, which can be transferred into a magnet, and works as electrical spin-orbit torque (SOT) to effectively manipulate its magnetization (see Figure 1a) [5,6,7]. By now, the electrical SOT has been intensively studied due to its essential role in the spin-orbitronic technology [8,9,10]. Recently, another class of the spin current, defined as magnon current, has emerged and attracted much attention [11,12,13,14]. For the magnon current, the spin angular momentum is carried by the precessing spin moments, which does not request movement of the electrons (see Figure 1b). Therefore, the Joule heat dissipation can be drastically reduced in the magnon-based spin-orbitronic devices. Very recently, Wang et al. reported the successful observation of the magnon torque transferred into a metallic magnet through an antiferromagnetic insulator, and they realized the magnetization switching of the metallic magnet by the magnon torque [13]. On the other hand, it has been known that the electrical SOT can be transferred into a magnetic insulator (MI) and drive its magnetization [15,16,17,18]. Therefore, it is natural to ask whether the magnon torque can be efficiently transferred into the MI, since it may provide a practical approach to further lower the energy dissipation in the spin-orbitronic devices. To study the magnon torque in the MI is important in both fundamental and practical aspects of spin-orbitronics.

In this work, we study the magnon torque transferred into the Tm${}_{3}$Fe${}_{5}$O${}_{12}$ (TmIG), a typical MI. By fabricating the Pt/NiO/TmIG heterostruture with different NiO thicknesses, we systematically investigate the evolution of the transferred magnon torque. We show that the magnon torque efficiency transferred through the NiO into the MI can retain a high value, which is comparable to the previous report for the magnon torque transferred into the metallic magnet.

## 2. Materials and Methods

For the sample fabrication, a 4-nm-thick TmIG film was deposited on Gd${}_{3}$Sc${}_{2}$Ga${}_{3}$O${}_{12}$ (GSGG) (111) single crystal substrates at 700 ${}^{{}^{\circ}}$C by magnetron sputtering. After cooling down to the room temperature, NiO films with thicknesses from 0 to 30 nm were deposited on the TmIG surface, and then a 4-nm-thick Pt film was deposited on the NiO surface. All the films were deposited without breaking the vacuum. The base pressure in the chamber before deposition was better than 1 × 10${}^{-6}$ Pa, and the deposition pressure was 0.4 Pa. The film thickness was controlled by the deposition time with a precalibrated deposition rate. All the measurements were conducted at room temperature.

## 3. Results and Discussion

**Figure 2:**
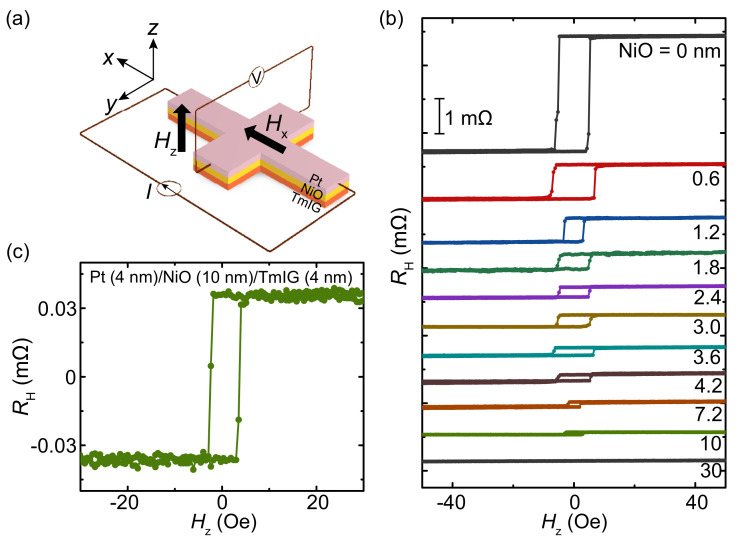
(**a**) Schematic of the setup for the AHE resistance measurement. (**b**) AHE resistance curves of the Pt (4 nm)/NiO (*t* nm)/TmIG (4 nm) devices with different NiO thicknesses. (**c**) AHE resistance curve of the Pt (4 nm)/NiO (10 nm)/TmIG (4 nm) device.

The anomalous Hall effect (AHE) was measured by patterning the Pt/NiO/TmIG films into Hall bar devices, as shown in Figure 2a. A constant charge current was injected into the films along the *x*-axis, and the Hall voltage was measured by sweeping an external magnetic field $H_{z}$ along the *z*-axis. Figure 2b exhibits the AHE resistance $R_{H}$ curves of the Pt (4 nm)/NiO (*t* nm)/TmIG (4 nm) devices with different NiO thicknesses. In the case of *t* = 0 nm, $R_{H}$ is obtained as about 1.7 mΩ. By increasing *t* to 0.6 nm, $R_{H}$ drastically decreases to about 0.5 mΩ. In the Pt/TmIG heterostructure, the anomalous Hall signal attributes to both the proximity-induced ferromagnetism of Pt and the spin current [19]. Therefore, although the TmIG is an insulator, the proximity-induced ferromagnetic layer in Pt can generate an AHE signal as normal ferromagnetic conductors do. Simultaneously, the spin current via the nonzero imaginary part of the spin-mixing conductance can also generate an AHE-like signal at the Pt/TmIG interface. By inserting the 0.6-nm-thick NiO layer, the proximity effect is eliminated. Furthermore, the ultrathin NiO layer without antiferromagnetic ordering only acts as an insulator, which prevents the spin transportation from the Pt to the TmIG layer. Both effects result in this drastic decrease of the AHE resistance. However, by further increasing the NiO thickness, although the proximity effect is completely eliminated, we still can observe the AHE signal. This is because, by increasing the NiO thickness, the long-range antiferromagnetic order restores, and then the magnon current can transport through the antiferromagnetic NiO layer [13]. As shown in Figure 2c, the AHE resistance curve of the Pt (4 nm)/NiO (10 nm)/TmIG (4 nm) device unambiguously confirms the magnon current transferred into the TmIG layer through the NiO layer.

**Figure 3:**
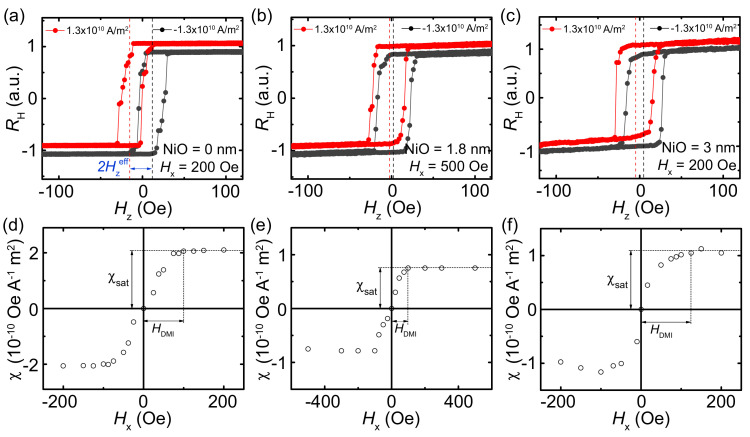
AHE resistance curves of the Pt (4 nm)/NiO (*t* nm)/TmIG (4 nm) devices by sweeping $H_{z}$ with (**a**) *t* = 0 nm and $H_{x}$ = 200 Oe, (**b**) *t* = 1.8 nm and $H_{x}$ = 500 Oe, (**c**) *t* = 3 nm and $H_{x}$ = 200 Oe. Slight vertical offsets for both AHE loops are introduced for clarity. Corresponding $H_{x}$ dependence of the SOT efficiency χ for (**d**) *t* = 0 nm, (**e**) *t* = 1.8 nm and (**f**) *t* = 3 nm. $\chi_{\mathrm{sat}}$ is the maximum SOT efficiency, and $H_{\mathrm{DMI}}$ is the field that χ saturates.

In the following, we measure the damping-like torque-induced effective field $H_{z}^{\mathrm{eff}}$ in the Pt (4 nm)/NiO (*t* nm)/TmIG (4 nm) devices with different NiO thicknesses. An external magnetic field along the *z*-axis $H_{z}$ was swept to measure the AHE with a constant external magnetic field along the *x*-axis $H_{x}$, as shown in Figure 2a. In the N$\overset{´}{e}$el-type domain walls, the damping-like torque works as an additional effective field along the *z*-axis, which favors one type of the domain [20,21]. This results in a horizontal shift of the AHE hysteresis loop. Figure 3a–c presents typical shifted hysteresis loops of the devices with different NiO thicknesses. By applying a positive current, the hysteresis loop shifts to the left, which is vice versa for a negative current. By measuring the current-induced shift $H_{z}^{\mathrm{eff}}$, the magnitude of the damping-like torque can be determined. Here, we define the damping-like SOT generation efficiency χ as χ = $H_{z}^{\mathrm{eff}}$/$J_{e}$, where $J_{e}$ is the applied current density. The corresponding $H_{x}$ dependence of χ are summarized in Figure 3d–f, respectively. First, χ gradually increases with $H_{x}$ and then saturates at a large in-plane field $H_{x}^{\mathrm{sat}}$. $H_{x}^{\mathrm{sat}}$ represents the minimum field to overcome the effective Dzyaloshinskii–Moriya interaction (DMI) field $H_{\mathrm{DMI}}$. Therefore, we can obtain the maximum SOT efficiency $\chi_{\mathrm{sat}}$ = 2.1 × 10${}^{-10}$ Oe A${}^{-1}$ m${}^{2}$ and $H_{\mathrm{DMI}}$ ≈ 120 Oe for NiO = 0 nm. For NiO = 1.8 nm, $\chi_{\mathrm{sat}}$ = 0.75 × 10${}^{-10}$ Oe A${}^{-1}$ m${}^{2}$ and $H_{\mathrm{DMI}}$ ≈ 100 Oe are obtained. For NiO = 3 nm, $\chi_{\mathrm{sat}}$ = 1.1 × 10${}^{-10}$ Oe A${}^{-1}$ m${}^{2}$ and $H_{\mathrm{DMI}}$ ≈ 125 Oe are obtained. This result shows that $\chi_{\mathrm{sat}}$ changes nonmonotonically with the NiO thickness.

**Figure 4:**
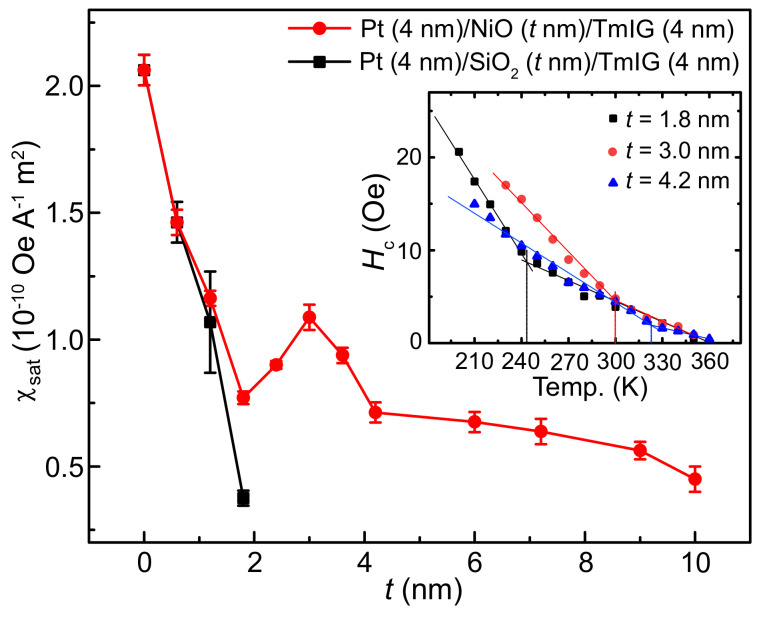
The dependence of the maximum SOT efficiency $\chi_{\mathrm{sat}}$ on the NiO thickness. Pt (4 nm)/SiO${}_{2}$ (*t* nm)/TmIG (4 nm) devices were measured for comparison. The inset shows the temperature dependence of the coercivity $H_{c}$ for the Pt (4 nm)/SiO${}_{2}$ (*t* nm)/TmIG (4 nm) devices.

Figure 4 summarizes the NiO thickness dependence of $\chi_{\mathrm{sat}}$. It can be seen that $\chi_{\mathrm{sat}}$ drastically decreases by increasing the NiO thickness to 1.8 nm. In this range, the antiferromagnetic ordering is weak due to the ultrathin NiO thickness, and the magnon torque only plays a minor role. The SOT in this range is due to the electron spin tunneling effect through the insulator. Above 1.8 nm, $\chi_{\mathrm{sat}}$ starts to increase, which unambiguously confirms the presence of the magnon torque, since the magnons in the NiO are the only spin-angular-momentum carriers. We also fabricated and measured the Pt (4 nm)/SiO${}_{2}$ (*t* nm)/TmIG (4 nm) devices for comparison. When SiO${}_{2}$ thickness is below 1.8 nm, it shows a similar decrease due to the electron spin tunneling effect, and no signal can be detected above 1.8 nm. For the Pt (4 nm)/NiO (*t* nm)/TmIG (4 nm) devices, a peak value of $\chi_{\mathrm{sat}}$ is obtained at *t* = 3 nm, which is about 50% of that for the Pt/TmIG device. Wang et al. reported the magnon torque transferred into the metallic magnet, and the peak value is about 45% of that for the control devices without NiO [13]. Our study demonstrates that the magnon torque transferred trough the NiO into the MI can also retain a high efficiency. By further increasing the NiO thickness up to 10 nm, the magnon torque generation can still be detected, although it gradually decreases due to the energy dissipation caused by magnon–magnon and magnon–phonon coupling. A previous study demonstrates that the spin fluctuation in the NiO layer can increase the spin conductivity [22]. Since the spin fluctuation can be enhanced near the N$\overset{´}{e}$el temperature, an enhancement of the SOT generation can be expected near the N$\overset{´}{e}$el temperature. In our study, in order to determine the N$\overset{´}{e}$el temperature, we measured the temperature dependence of the coercivity $H_{c}$ for *t* = 1.8, 3 and 4.2 nm, respectively. When the temperature is below the N$\overset{´}{e}$el temperature, an abrupt enhancement is expected to occur due to the presence of the ferromagnetic-antiferromagnetic interaction [23]. As shown in the inset of Figure 4, the N$\overset{´}{e}$el temperature is about 250 K for *t* = 1.8 nm, 300 K for *t* = 3 nm and 325 K for *t* = 4.2 nm. Therefore, the sharp peak of $\chi_{\mathrm{sat}}$ at *t* = 3 nm is attributed to the enhancement of the spin fluctuations in the NiO layer.

We notice that in Wang et al.’s study, the peak value of the magnon torque efficiency appears when the NiO thickness is 25 nm, which is thicker than that in our study (3 nm) [13]. This could be caused by the different NiO quality grown with different deposition conditions.

## 4. Conclusions

In summary, we have demonstrated the magnon torque transferred into an MI through an antiferromagnetic insulator. By fabricating the Pt/NiO/TmIG heterostructure with different NiO thicknesses, we have systematically investigated the evolution of the magnon torque efficiency. We show that the magnon torque transferred through the NiO into the MI can retain about 50%, which is comparable to the previous report for the magnon torque transferred into the metallic magnet. Our study manifests the feasibility of realizing the pure magnon-based spintronic devices with ultralow energy consumption and high efficiency.

## Data Availability

All the data present in this paper will be made available upon reasonable request. Please contact the corresponding author for further information.
